# Loss of p38δ mitogen-activated protein kinase expression promotes oesophageal squamous cell carcinoma proliferation, migration and anchorage-independent growth

**DOI:** 10.3892/ijo.2013.1968

**Published:** 2013-05-29

**Authors:** CAROL O’CALLAGHAN, LIAM J. FANNING, AILEEN HOUSTON, ORLA P. BARRY

**Affiliations:** 1 Departments of Pharmacology and Therapeutics, University College Cork, Ireland; 2 Medicine, University College Cork, Ireland

**Keywords:** oesophageal cancer, p38δ mitogen-activated protein kinase, proliferation, migration, anchorage-independent growth

## Abstract

Oesophageal cancer is an aggressive tumour which responds poorly to both chemotherapy and radiation therapy and has a poor prognosis. Thus, a greater understanding of the biology of oesophageal cancer is needed in order to identify novel therapeutic targets. Among these targets p38 MAPK isoforms are becoming increasingly important for a variety of cellular functions. The physiological functions of p38α and -β are now well documented in contrast to -γ and -δ which are comparatively under-studied and ill-defined. A major obstacle to deciphering the role(s) of the latter two p38 isoforms is the lack of specific chemical activators and inhibitors. In this study, we analysed p38 MAPK isoform expression in oesophageal cancer cell lines as well as human normal and tumour tissue. We observed specifically differential p38δ expression. The role(s) of p38δ and active (phosphorylated) p38δ (p-p38δ) in oesophageal squamous cell carcinoma (OESCC) was delineated using wild-type p38δ as well as active p-p38δ, generated by fusing p38δ to its upstream activator MKK6b(E) via a decapeptide (Gly-Glu)_5_ linker. OESCC cell lines which are p38δ-negative (KE-3 and -8) grew more quickly than cell lines (KE-6 and -10) which express endogenous p38δ. Re-introduction of p38δ resulted in a time-dependent decrease in OESCC cell proliferation which was exacerbated with p-p38δ. In addition, we observed that p38δ and p-p38δ negatively regulated OESCC cell migration *in vitro*. Finally both p38δ and p-p38δ altered OESCC anchorage-independent growth. Our results suggest that p38δ and p-p38δ have a role in the suppression of OESCC. Our research may provide a new potential target for the treatment of oesophageal cancer.

## Introduction

Oesophageal cancer is the seventh most common cancer worldwide (1) with its 5-year survival rate being dismally low at ≤15% (2). Oesophageal squamous cell carcinoma (OESCC) is an exceptionally drug-resistant tumour. Despite recent advances in the detection of OESCC and the development of multimodal therapy (3,4), its incidence is on the rise and outcome for patients remains poor (5,6). Thus, a greater understanding of the initiation and progression of OESCC is required in order to be able to identify predictive and prognostic factors that may in the future lead to novel therapeutic strategies.

The mitogen-activated protein kinases (MAPKs) are serine/threonine kinases and include the extracellular-regulated kinase (ERK), c-jun NH_2_-terminal kinase (JNK) and p38 MAPK families. The p38 MAPK family consists of four members; p38α (MAPK14) of which there are two splice variants (7), p38β (MAPK11), p38γ (MAPK12) and p38δ (MAPK13) (8). Although these isoforms are 60–70% identical in amino acid sequence they differ greatly in their tissue distribution (9), substrate specificity (10) and sensitivity to chemical inhibitors (11). In recent years, we have gained an increased appreciation of the importance of p38 isoforms for a variety of cellular functions including proliferation, differentiation, transformation and programmed cell death (12). Their roles, however, are more complex than previously thought, with distinct members appearing to have different functions. In addition, the roles of p38 in various pathologic conditions remain to be elucidated (13).

To-date most of the published literature refers to the p38 family as a whole or indeed have focused on the first discovered isoform p38α (10,13). There is an obvious dearth of research pertaining to the latter two isoforms, p38γ and -δ, due partly to the lack of commercially available specific chemical activators or inhibitors for each of these isoforms (14). In the present study we have overcome this obstacle using an enzyme-substrate fusion approach for the generation of constitutively active p38δ. We now provide new information regarding the role(s) of p38δ and active (phosphorylated) p38δ (p-p38δ) in OESCC. We identified differential p38δ expression in OESCC. Lack of p38δ expression in OESCC allows for a more aggressive phenotype including increased proliferation, increased migration and increased capacity for anchorage-independent growth. Restoration of p38δ expression, however, reverses these effects. Together, our results provide evidence for a novel role for p38δ-induced suppressive effects in OESCC. With survival rates being poor for patients with OESCC, there is an urgent need to find novel strategies to improve current therapy. Our study suggests isoform specific activation of p38δ as a possible potential approach for treatment of patients with OESCC.

## Materials and methods

### Reagents

All chemicals and cell culture reagents were purchased from Sigma-Aldrich (Wicklow, Ireland), enzymes from New England BioLabs (Hertfordshire, UK) and primary antibodies from Cell Signaling Technologies (Hertfordshire, UK), unless otherwise stated.

### Specimens

The patient cohort consisted of ten patients with OESCC of both genders ranging in age from 44 to 81 years. Formalin-fixed, paraffin-embedded (FFPE) oesophagectomy specimens from ten patients consisted of ten paired samples of primary tumour and metastatic lymph nodes with 10 samples of non-tumour adjacent tissues (NAT). Patient features are summarized in Table IA.

### Cell culture

The KE oesophageal cancer cell lines (kind gifts from Professor T. Fujii, Kurume University School of Medicine, Japan) (15–17) as well as KYSE-70, OE-19, OE-21 and OE-33 (ATCC, Rockville, MD, USA) were cultured in RPMI-1640 supplemented with 10% FCS, 100 *μ*g/ml streptomycin and 100 U/ml penicillin. KE cell line features are summarized in Table IB. The metastatic oesophageal cancer cell line, OC-3 [a kind gift from Cork Cancer Research Centre, (Biosciences Institute, National University of Ireland, Cork, Ireland] (18) was cultured in DMEM supplemented with 10% FCS, 100 *μ*g/ml streptomycin and 100 U/ml penicillin. KYSE-450 cells (ATCC, Rockville, MD, USA) were maintained in 45% RPMI-1640/45% Ham’s F-12 nutrient mixture supplemented with 10% FCS, 100 *μ*g/ml streptomycin and 100 U/ml penicillin.

### Proliferation assay

KE cells were plated at a density of 3×10^4^ cells/well in a 6-well tissue culture plate. Cell viability was assessed by trypan blue (0.4% w/v) exclusion assay at the indicated times (18).

### Nuclear and cytosolic extraction

Nuclear and cytosolic fractions were isolated from 2×10^6^ cells using the NE-PER Isolation kit (Pierce Biotechnology, Rockford, IL, USA) according to the manufacturer’s instructions.

### Generation of MKK6b-p38δ MAPK, MKK6b(E)-p38δ MAPK and MKK6b(E)-p38δ_DN_ MAPK fusion proteins

p38δ (pcDNA3-FLAG-p38δ) and constitutively active MKK6b [pcDNA3-MKK6b(E)] plasmids were a kind gift from Professor J. Han (Scripps Research Institute, La Jolla, CA, USA) and have previously been described (19). To construct the pcDNA3-MKK6b(E)-FLAG-p38δ (p-p38δ) fusion plasmid the TAA stop codon of MKK6b(E) was replaced with a unique *Swa*I restriction sequence using a QuikChange Lightening Site-Directed Mutagenesis kit (Agilent Technologies) (5′-CAT CTTTTGTAAAACTGATTCTTGGAGAATTTAAATCAG TGGACTTAATCGGTTGACCCTACTG-3′; 5′-CAGTAG GGTCAACCGATTAAGTCCACTGATTTAAATTCTCCAA GAATCAGTTTTACAAAAGATG-3′). A PCR generated *Dra*I-*Dra*I fragment encoding FLAG-p38δ with a 5′ (Gly-Glu)_5_ linker (5′-CCGCGCTTTAAAGGCGAGGGCGAGGG CGAGGGCGAGGGCGAGATGGACTACAAGGACGAC GAT-3′; 5′-TTGATCTTTAAATTATTACAGCTTCATG CCACTTCGT-3′) to facilitate folding as previously described (20) was ligated to *Sw*aI linearised pcDNA3-MKK6b(E) with T4 DNA ligase. The pcDNA3-MKK6b-FLAG-p38δ plasmid (inactive MKK6b) was created by the substitution of Glu^151^ and Glu^155^ with Ser and Thr respectively by site-directed muta-genesis (5′-TGGAATCAGTGGCTATTTGGTGGACTCTGT TGCTAAAACAATTGATGCAGGTTGCAAACCATAC-3′; 5′-GTATGGTTTGCAACCTGCATCAATTGTTTTAGCAA CAGAGTCCACCAAATAGCCACTGATTCCA-3′). The pcDNA3-MKK6b(E)-FLAG-p38δ_DN_ (dominant negative) (p-p38δ_DN_) plasmid was created by substituting Thr^180^ and Tyr^182^ of p38δ with Ala and Phe respectively by site-directed mutagenesis (5′-GACGCCGAGATGGCTGGCTTCGTGG TGACCCG-3′; 5′-CGGGTCACCACGAAGCCAGCCAT CTCGGCGTC-3′). DNA sequence analysis confirmed the integrity of all plasmids.

### Stable transfection

KE-3 cells were transfected using Lipofectamine™ 2000 reagent (Life Technologies™) and a total of 4 *μ*g of plasmid DNA according to the manufacturer’s instructions. Twenty-four hours following transfection cells were transferred to 100-mm diameter dishes and transfected cells were selected in growth medium containing 800 *μ*g/ml Geneticin. After 4–8 weeks, individual cell colonies were transferred for clone expansion.

### Immunoblot analysis

Supernatants used for immunoblotting with specific antibodies, p38α and -δ, phospho-p38 MAPK and MKK6 antibodies (New England Biolabs), p38γ (Upstate) and p38β_2_ antibody (Zymed Laboratories Inc.) have previously been described by us (18,21). Chemiluminescent detection was performed using SuperSignal^®^ WestDura Extended Duration Substrate (Pierce Biotechnology) and bands were visualized using a Syngene G:Box ChemiXR5 Gel Documentation System.

### Immunohistochemistry

This was performed as previously described by us (21). Briefly, FFPE OESCC and NAT sections were de-parrafinized in xylene and re-hydrated prior to analysis. Antigen retrieval was performed by microwave irradiation in 0.01 M citrate buffer, pH 6.0. In addition cultured cells grown on coverslips were fixed in 2-4% paraformaldehyde and permeabilised with 0.5% Triton-X-100. Samples were blocked with 5% NGS in TS/SAP. Slides were incubated with primary antibody overnight at 4°C. Antibody binding was localized using a biotinylated secondary antibody, avidin-conjugated HRP and DAB substrate, contained within the Vectastain ABC detection kit (Vector Laboratories, Burlingame, CA, USA). Slides were counterstained with hematoxylin.

### ELISA

Cell lysates were analysed for p38δ phosphorylation at T180/Y182 using the R&D Systems DuoSet^®^ IC Human phospho-p38δ (T180/Y182) sandwich ELISA (DYC2124-5) according to the manufacturer’s instructions. Absorbance was read at 450 nm on a Tecan Sunrise spectrophotometric plate reader and analysed using the XRead software program.

### Boyden chamber cell migration assay

Cells were plated in starvation medium at a density of 3×10^4^ cells/well into a 96-well plate of the upper chamber. The bottom chamber contained 10% FCS as the chemoattractant. Cells were left migrate for 24 h through the matrigel filter (8 mm). Migrated cells were treated with MTT (3-(4,5-dimethylthiazol-2-yl)-2,5-diphenyltetrazolium bromide) (5 mg/ml) and absorbance read at 540 nm to calculate viable cell numbers as previously described (21).

### Wound-healing assay

Cell migration was assessed by *in vitro* wound-healing assay as previously described (22). A linear wound track was made by use of a sterile tip through confluent cells. Cells migrating into the wound were captured under a phase-contrast microscope 24 and 48 h after wounding. Migration was determined using the ImageJ program as an average closed area of the wound relative to the initial wound area at 24 and 48 h after wounding.

### Colony forming assay

The role of p38δ in anchorage-independent growth was assayed using a soft agar colony-forming assay as previously described (21). Cells were plated at a density of 3×10^5^ cells/100-mm dish in medium containing 0.4% (w/v) agar on an underlay of 0.8% (w/v) agar. After a 21-day incubation colonies were stained with MTT (5 mg/ml) overnight and counted.

### siRNA

KE-6 cells at 75% confluency in antibiotic-free media were transfected with 100 nM p38δ MAPK siRNA or control siRNA-A (Santa Cruz Biotechnology, Santa Cruz, CA, USA) according to the manufacturer’s instructions and as recently described (23).

### RT-PCR

First-strand cDNA was synthesised using SuperScript^®^ VILO™ cDNA Synthesis kit (Life Technologies) from total RNA isolated from cells using an Illustra RNASpin Mini kit (GE Healthcare, Buckinghamshire, UK) according to the manufacturer’s instructions. p38δ mRNA was amplified from cellular cDNA under the following conditions: ddH_2_O, 1X DreamTaq buffer, 0.2 mM dNTPs, 0.25 *μ*M p38δ forward primer: 5′-CCACGTTAAACTGCCCATCT-3′, 0.25 *μ*M p38δ reverse primer: 5′-CCGCCACAAGCTAAAAAGAG-3′, 1 *μ*l cDNA and 1 U DreamTaq DNA polymerase (Thermo Fisher Scientific; Waltham, MA, USA). RT-PCR products were analysed by agarose gel electrophoresis.

### Proteome Profiler™ antibody array

The relative levels of phosphorylation of 26 kinases was examined in cell lysates using a Proteome Profiler Human Phospho-MAPK array (R&D Systems, Abingdon, UK) according to the manufacturer’s instructions. Following chemiluminescent detection, pixel density of each spot was analysed using Scion image software.

### Ethics

The research was approved by the Clinical Research Ethics Committee of the Cork Teaching Hospitals.

### Statistical analysis

Results are expressed as mean ± SE. Statistical comparisons were made by using analysis of variance with subsequent application of Student’s t-test, as appropriate. GraphPad InStat 3 software was used also for statistical analysis.

## Results

### p38α, -β, -γ and -δ isoforms and MKK3, -4, -6 and 7 are differentially expressed in oesophageal cancer

**Figure 1:**
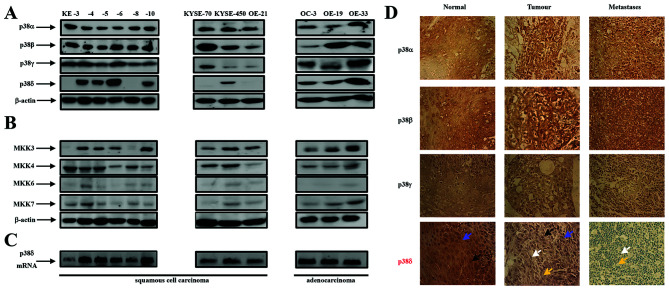
Expression of p38 MAPK isoforms, MKK3, -4, -6 and -7 in oesophageal cancer. (A) Western blot analysis of p38 isoform expression in KE-3, -4, -5, -6, -8 and -10, KYSE-70, -450 and OE-21 (oesophageal squamous cell carcinoma cell lines) as well as OC-3, OE-19 and OE-33 (oesophageal adenocarcinoma cell lines). (B) Western blot analysis of MKK3, -4, -6 and -7 in the same twelve cell lines. Aliquots of 30 *μ*g of protein lysate were loaded on a 10% SDS-PAGE gel and analyzed by immunoblotting using antibodies specific for p38α, -β_2_, -γ and -δ. β-actin analysis served as a loading control. The results shown are representative of four independent experiments. (C) Agarose gel electrophoresis analysis of DNA fragments produced by PCR amplification of p38δ mRNA from oesophageal squamous (KE3, -4, -5, -6, -8, 10, KYSE70, -450 and OE21) and adenocarcinoma (OC3, OE19 and -33) cell lines. (D) Immunohistochemical staining of p38α, -β_2_, -γ and -δ isoforms in normal, tumourigenic and metastatic (lymph node) oesophageal human tissue. Immunohistochemical staining was performed as outlined in Materials and methods. Blue arrow indicates cytoplasmic staining; black arrow indicates nuclear staining; white arrow indicates blue unstained nuclei and yellow arrow indicates blue unstained cytoplasm. Magnification, ×400. The results shown are representative of ten patients.

The expression of p38 as a family has previously been outlined in oesophageal cancer as well as other cancer types (10,13,24,25). While these reports refer to the p38 family, analysis of individual p38 isoform expression in oesophageal cancer has to date never been reported. A previous study by us outlining differential p38 isoform expression in renal cancer prompted us to investigate further the effects of individual p38 family members in cancer in general (26). Using western blot analysis we examined p38 MAPK isoform expression in nine OESCC cell lines (KE-3, -4, -5, -6, -8, -10, KYSE-70, KYSE-450 and OE-21) and three oesophageal adenocarcinoma cell lines (OC-3, OE-19 and OE-33). We used antibodies specific for each isoform p38α, -β_2_, -γ and -δ as previously described by us (26). All twelve oesophageal cancer cell lines (squamous and adenocarcinoma) expressed p38α, -β and -γ (albeit at different levels) (Fig. 1A). In contrast p38δ expression was present in the three adenocarcinoma cell lines but absent in four of the OESCC cell lines KE-3, -8, KYSE-70 and OE-21 (Fig. 1A). The specific loss of p38δ isoform expression only has previously been reported by us in renal carcinoma (786-0) (26) and also observed by us in liver (Huh-7), lung (A-549) prostate (PC-3 and DU-145) and skin (MeWo) cancer cell lines (Barry *et al*, unpublished data). Upstream MKK3 and -6 are thought to be the major protein kinases responsible for p38 activation (24) but the selectivity of p38 isoform activation is stimulus type and strength dependent (27). We observed strong MKK3 and -4 expression for all cell lines except KE-3 and -8 OESCC which were MKK3 negative. In contrast levels of MKK6 and -7 expression were considerably lower (Fig. 1B).

Finally, analysis of p38δ at the mRNA level surprisingly proved positive for all cell lines examined including the four OESCC cell lines that were negative for p38δ protein expression (Fig. 1C). Primers specific for a 292-bp fragment of the 3′-untranslated region of p38δ mRNA amplified cDNA from all twelve cell lines. Other primer sets within the coding sequence yielded similar results (data not shown). In addition DNA sequence analysis of PCR products did not identify any mutations such as a stop codon or a missense mutation which could possibly explain loss of p38δ protein expression (data not shown).

To investigate whether the p38 isoform expression pattern we observed *in vitro* with the OESCC cell lines could be translatable to the *in vivo* situation we analyzed the expression profile and localization of all four p38 isoforms (α, -β, -γ and -δ) in FFPE oesophagectomy specimens from ten patients with squamous cell carcinoma. Samples consisted of ten paired primary tumour and metastatic (lymph nodes) as well as corresponding non-tumour adjacent tissues (NAT) as outlined in Table IA. Samples were staged according to the new TNM7 categorization for oesophageal cancer (Table IA) (28). Consistent levels of p38α and -β expression was evident in all ten normal, primary and metastatic OESCC samples (Fig. 1D). Similarly, we did not observe a change in p38γ expression between normal, primary tumour and metastatic samples albeit the intensity of brown staining was less than that observed for p38α and -β (Fig. 1D). p38δ expression, however, was considerably different in normal vs primary tumour vs metastatic disease (Fig. 1D and Table IC). p38δ expression was observed in both the nuclei and cytoplasm of nine of the ten oesophageal NAT tissue samples. However, a significant decrease in expression was observed in both the nuclei and cytoplasm in the ten primary tumour specimens as evidenced from the lighter brown staining compared to NAT samples in six patient samples and complete loss of expression in four of the samples (Fig. 1D and Table IC). Furthermore, eight out of the ten metastatic tissue specimens demonstrated complete loss of p38δ expression with both the nuclei and cytoplasm appearing blue in colour (Fig. 1D). This is an important finding considering identification of lymph node metastasis is the single most important prognostic factor in oesophageal cancer (1).

### OESCC cell lines lacking endogenous p38δ MAPK expression proliferate faster than those which express this isoform

**Figure 2:**
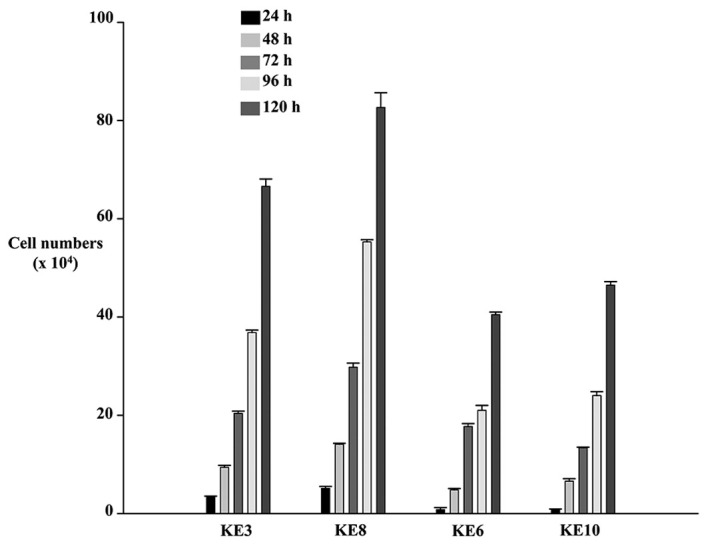
Oesophageal squamous cell carcinoma cell lines lacking endogenous expression of p38δ MAPK have a higher proliferation rate. KE-3 and -8 cell lines (lacking endogenous p38δ expression) and KE-6 and -10 cell lines (expressing endogenous p38δ expression) were seeded (3×10^4^) and counted for 24–120 h. The results shown are mean ± SE of three independent experiments.

The results obtained for differential p38δ expression in both the oesophageal cell lines and the human samples prompted us to investigate further the effect(s) if any this particular isoform may have on the tumourigenicity of OESCC. Firstly, we examined whether the absence or presence of endogenous p38δ expression could have an effect on the proliferation rate of our OESCC cell lines. Using the trypan blue exclusion assay we compared the proliferation rate of KE-3 and -8 cell lines (which do not express p38δ) versus KE-6 and -10 (which express p38δ). We observed that at all time-points studied (24–120 h) both cell lines KE-3 and -8 proliferated faster than KE-6 and -10 cells (Fig. 2).

### Generation of active (phosphorylated) p38δ (p-p38δ) MAPK fusion proteins

**Figure 3:**
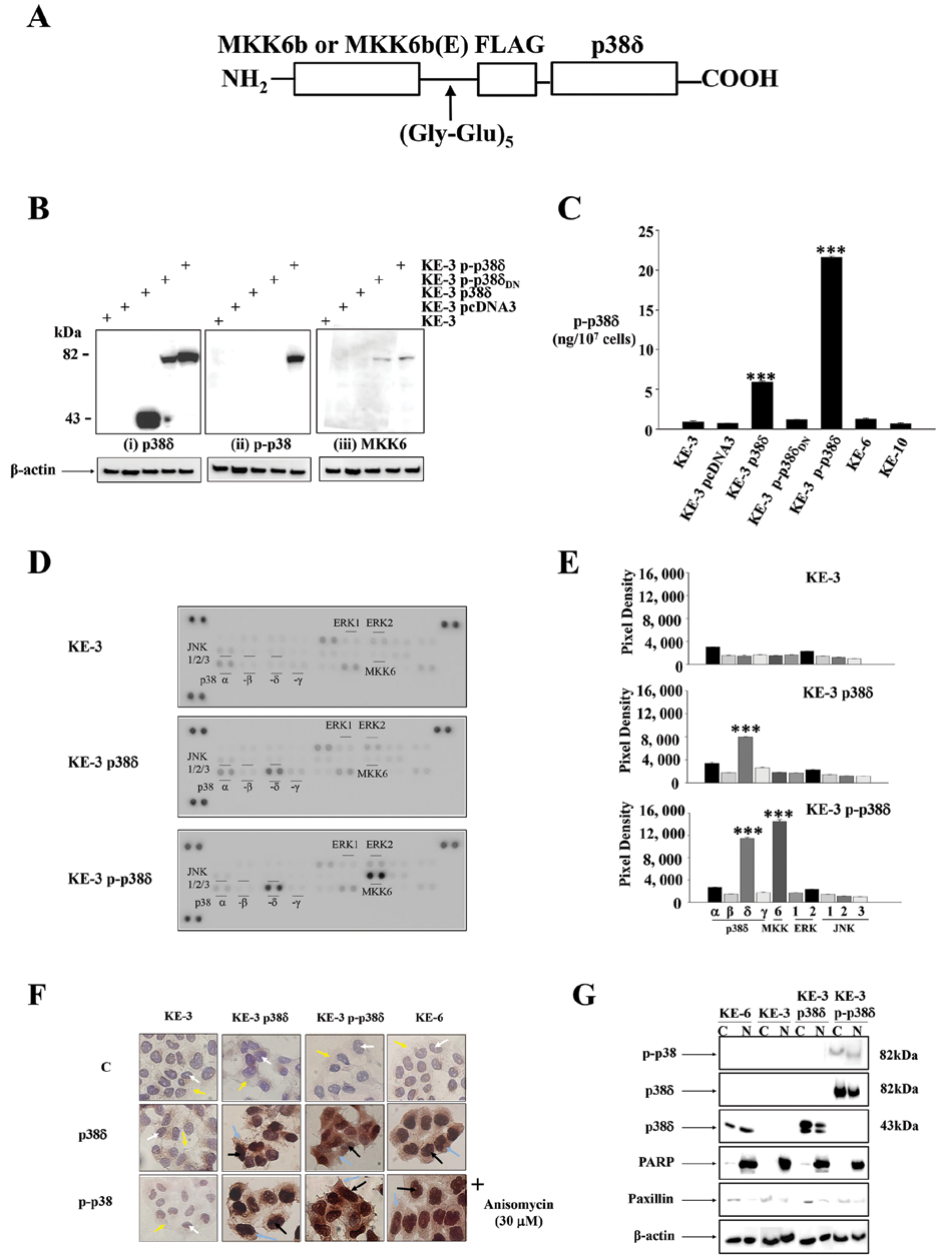
Generation of active p38δ (p-p38δ) MAPK fusion proteins. (A) A schematic representation of MKK6b-p38δ or MKK6b(E)-p38δ MAPK fusion protein. The coding region of p38δ was fused in frame to the 3′-end of the stop codon-less MKK6b or MKK6b(E) through a peptide linker (Gly-Glu)_5_. (B) Western blot analysis of KE-3 cells stably transfected with empty vector (pcDNA3), wild-type p38δ, p-p38δ and p-p38δ_DN_. Cells were analysed by immunoblot using antibodies specific for p38δ (i), p-p38 (ii) and MKK6 (iii). Aliquots of 30 *μ*g protein lysate for each cell line were loaded on a 10% SDS-PAGE gel. The results shown are representative of four independent experiments. (C) Transfected and non-transfected KE-3, KE-6 and KE-10 cells were analysed to determine the amount of activated i.e., phosphorylated p38δ expression using the human phospho-p38δ (T180/Y182) ELISA commercial kit (R&D Systems). The ELISA assay was carried out according to the manufacturer’s protocol. The results shown are mean ± SE of three independent experiments. Significant (***p<0.001) changes from control non-transfected KE-3 cells. (D) The human phospho-MAPK array shows the effects of stably transfecting KE-3 cells with p38δ and p-p38δ. Arrays were incubated with 200 *μ*g of cell lysate. (E) Corresponding pixel density for p38α, -β, -δ and -γ, MKK-6, ERK1/2 and JNK1/2/3 phosphorylation in non-transfected and transfected KE-3 cells. (F) Immunohistochemical subcellular localization of p38δ and p-p38 in KE-3 non-transfected cells and cells transfected with p38δ and p-p38δ. KE-6 cells were or were not treated with anisomycin (30 *μ*M). (G) Nuclear and cytoplasmic localization of p38δ and p-p38 in KE-3 and KE-6 cells. The results shown are representative of four independent experiments (F and G).

To investigate whether p38δ or active (phosphorylated) p38δ (p-p38δ) drives the observed anti-proliferative phenotype (Fig. 2) we re-introduced wild-type p38δ into KE-3 cells which have lost its expression. In the absence of a specific commercially available p38δ activator [and to investigate the effect(s) of active (p-p38δ)] we generated a constitutively active p38δ through enzyme substrate fusion as previously described for JNK (Fig. 3A) (20). Western blot analysis of stable transfections of KE-3 cells demonstrated that pcDNA3-MKK6b-(Gly-Glu)_5_-FLAG-p38δ (data not shown) as well as pcDNA3-MKK6b(E)-(Gly-Glu)_5_-FLAG-p38δ both produced a single polypeptide with a molecular mass of 82 kDa as expected when using p38δ, p-p38 and MKK6 antibodies, respectively (Fig. 3B). As both MKK6b and MKK6b(E) fused in frame to p38δ produced the same desired result only one plasmid (MKK6b(E)-p38δ) was used for subsequent experiments. Western blot analysis of KE-3 cells stably transfected with pcDNA3-MKK6b(E)-(Gly-Glu)_5_-FLAG-p38δ_DN_ also produced a single polypeptide with a molecular mass of 82 kDa upon incubation with p38δ and MKK6 antibodies (Fig. 3Bi and iii) but did not demonstrate p38 activation (phosphorylation) (Fig. 3Bii). Of note the antibody used in Fig. 3Bii is a pan phospho-p38 antibody. To our knowledge there is no commercially available antibody to test for active (phosphorylated) p-p38δ specifically by western blot analysis. Therefore, to confirm p38δ activation we performed a sandwich ELISA which measures p38δ isoform phosphorylation specifically. Transfection of KE-3 cells with wild-type p38δ alone revealed activation (Fig. 3C). This is in strong agreement with previous reports where adenovirally expressed wild-type p38δ was activated in head and neck squamous cell carcinoma (29) and human keratinocytes (30). A 4-fold (p<0.001) increase in activation of p38δ was observed following stable transfection of KE-3 cells with p-p38δ (Fig. 3C). This level of activation is similar to KE-3 p38δ transfected cells upon activation with anisomycin (30 *μ*M) (data not shown). As expected we did not observe phosphorylation of p38δ in cells transfected with p-p38δ_DN_ (Fig. 3C). We also analysed KE-6 and KE-10 cell lines (which express endogenous p38δ expression) but did not observe p38δ phosphorylation in either cell line (Fig. 3C).

To ensure specific phosphorylation of p38δ only and not the other three p38 isoforms (α, -β and -γ) we performed a human phospho-MAPK antibody array (R&D Systems). We did not observe phosphorylation of p38α, -β or -γ in nontransfected KE-3 cells or cells stably transfected with p38δ or p-p38δ (Fig. 3D and E). We did, however, observe an increase (p<0.001) in phosphorylation in KE-3 p38δ wild-type transfected cells which was amplified in KE-3 p-p38δ transfected cells (Fig. 3D and E) in agreement with our ELISA results (Fig. 3C). These results confirm phosphorylation of p38δ only in our studies. We also observed MKK6 phosphorylation in KE-3 p-p38δ as expected (Fig. 3D and E). A previous report outlined p38δ induced inactivation of ERK1/2 (31) however, we did not find any change in ERK1/2 or indeed JNK1/2/3 (Fig. 3D and E).

Finally, the physical location of a protein either in the nucleus or the cytoplasm directly influences its biological function. Members of the p38 family do not contain either a nuclear localisation signal (NLS) or a nuclear export signal (NES) but their subcellular localisation can be regulated in part by their interacting proteins (32). We compared the subcellular localization of p38δ and p-p38 in KE-3 transfected cells with endogenous p38δ expression in KE-6 cells. As expected p38δ and p-p38 were absent from both compartments in non-transfected KE-3 cells (Fig. 3F). p38δ and p-p38 were detected in both the cytoplasm and the nucleus of KE-3 stably transfected cells (Fig. 3F). This pattern of expression correlated with the subcellular localization of p38δ and p-p38 in KE-6 cells in the presence and absence of anisomycin (30 *μ*M). To confirm our immunohistochemical findings cytosolic and nuclear extracts were prepared from transfected and non-transfected KE-3 and KE-6 cells and examined by western blot analysis. The use of PARP as a nuclear-restricted marker and Paxillin as a cytosolic marker ensured that there was no cross contamination between the subcellular fractions (21). Similar results were observed demonstrating the presence of p38δ and p-p38 in both the cytoplasm and nucleus of KE-3 and -6 cells (Fig. 3G).

### KE-3 cells transfected with p38δ and p-p38δ MAPK show reduced proliferation

**Figure 4:**
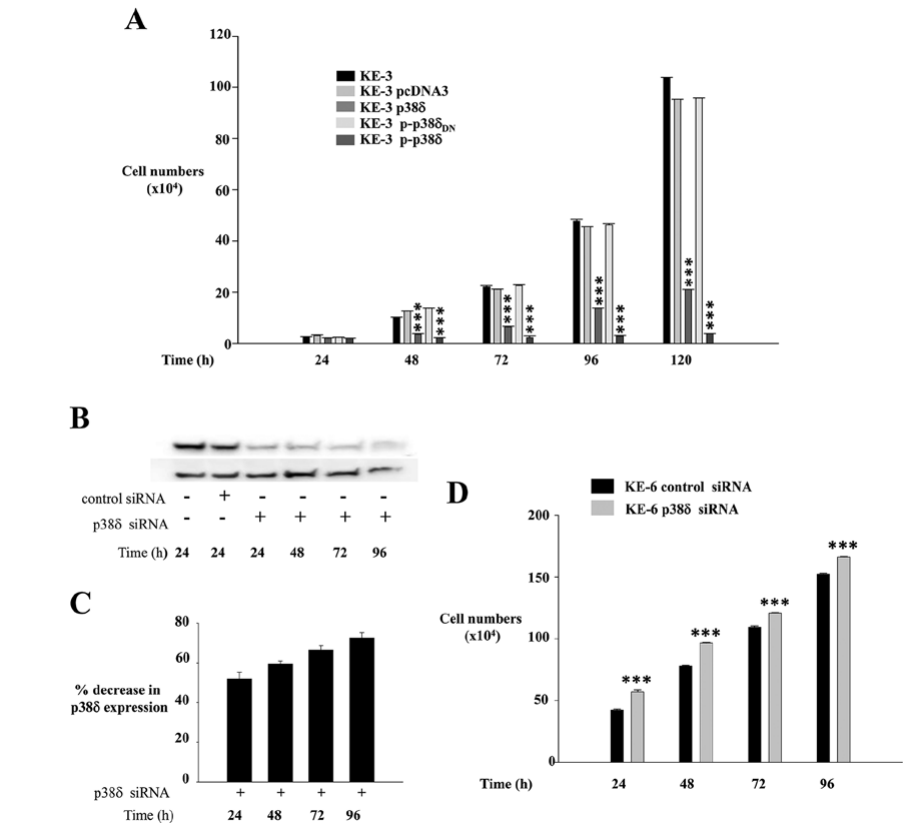
Effect of p38δ and p-p38δ MAPK expression on cell proliferation. (A) KE-3, KE-3 pcDNA3, KE-3 p38δ, KE-3 p-p38δ and KE-3 p-p38δ_DN_ cells were seeded (3×10^4^) and counted for 24–120 h. The results shown are mean ± SE of three independent experiments. Significant (^***^p<0.001) changes from control non-transfected KE-3 cells. (B) Western blot analysis of KE-6 cells transiently transfected or not transfected with p38δ siRNA or control siRNA for 24–96 h. Cells were analysed by immunoblotting using a p38δ antibody. Aliquots of 30 *μ*g protein lysate were loaded on a 10% SDS-PAGE gel. The results shown are representative of three independent experiments. (C) Densitometric analysis was performed to analyse % knockdown of KE-6 p38δ protein. (D) KE-6 cells (3×10^4^) transfected with p38δ siRNA or control siRNA were seeded and counted for 24–96 h. The results shown are mean ± SE of three independent experiments each done in triplicate. Significant (^***^p<0.001) changes from control siRNA transfected KE-6 cells.

Uncontrolled cellular proliferation is a hallmark of cancer. To investigate if loss of p38δ expression specifically drives the higher growth kinetics observed in Fig. 2 we compared the growth rates of KE-3 non-transfected and transfected cells. We observed a significant (p<0.001) time-dependent decrease in the proliferation rate of KE-3 cells when transfected with wild-type p38δ compared with nontransfected cells and cells transfected with empty pcDNA3 vector (Fig. 4A). This anti-proliferative effect was amplified further in KE-3 cells transfected with active p-p38δ (Fig. 4A). KE-3 cells transfected with p-p38δ_DN_ demonstrated the same proliferation rate as non-transfected cells or cells transfected with pcDNA3 only (Fig. 4A).

To further examine the hypothesis that p38δ is anti-proliferative in OESCC we employed a siRNA approach using the KE-6 cell line which expresses endogenous p38δ (Figs. 1, 3F and G). KE-6 cells were transiently transfected with p38δ siRNA or control siRNA as previously described (23). We observed a 51.9±6.5% reduction in KE-6 p38δ expression at 24 h following p38δ siRNA transfection which increased to 72.6±2.6% by 96 h when compared to control siRNA transfected KE-6 cells (Fig. 4B and C). No change in p38δ expression was observed when KE-6 cells were transfected with control siRNA for all time-points studied (24–96 h) (only 24 h is shown in Fig. 4B). A significant (p<0.001) increase in cell proliferation was observed for KE-6 cells transfected with p38δ siRNA compared to cells transfected with control siRNA for all time-points studied (Fig. 4D). The anti-proliferative effect was observed even in the absence of active p38δ in KE-6 cells (Fig. 3C). This effect on proliferation may be independent of its kinase activity as has previously been reported for p38α in regulating HeLa cell proliferation (33) and p38γ in rat intestinal epithelial cells (34).

### p38δ and p-p38δ MAPK play a role in migration and anchorage-independent growth of KE-3 cells

**Figure 5:**
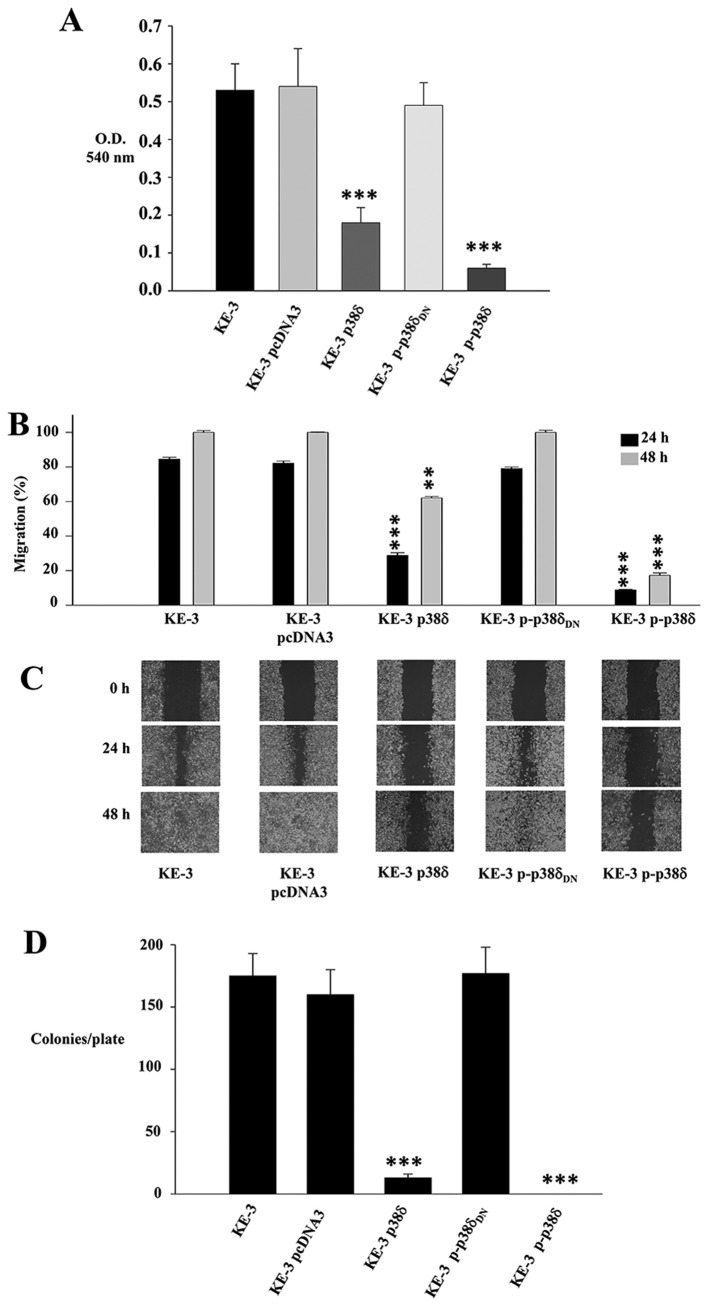
Effect of p38δ and p-p38δ MAPK on KE-3 cell migration and anchorage-independent growth. KE-3, KE-3 pcDNA3, KE-3 p38δ, KE-3 p-p38δ and KE-3 p-p38δ_DN_ cells were analysed for cell migration (A–C) and anchorage-independent growth D). (A and B) p38δ and p-p38δ inhibit KE-3 migration at 24 h (A, Boyden Chamber) and 24 and 48 h (B, wound healing). (C) Representative wound healing images at 0, 24 and 48 h. Wound healing rates decrease in p38δ and p-p38δ transfected KE-3 cells. The results shown are representative of three independent experiments. (D) Anchorage-independent growth potential of KE-3 non-transfected and transfected cells were measured by their ability to form colonies on soft agar. Plates were stained with 3-(4,5-dimethylthiazol-2-yl)-2, 5-diphenyl tetrazolium bromide to visualize colonies. The number of colonies per plate is shown. The results shown are mean ± SE of four independent experiments (A, B and D). Significant (^**^p<0.01; ^***^p<0.001) changes from control non-transfected KE-3 cells.

A key characteristic of cancer cells is their ability to migrate and progress from primary tumours to metastases in distant organs. A recent report summarizes the roles of p38 MAPKs in cancer invasion and metastasis (35). This review, however, as in previous reports documents the roles of p38 family as a whole or p38α (10,13). We examined the role of p38δ in OESCC cell migration using both a Boyden chamber assay and a wound healing assay. We observed a 66±7.5 and 88.7±1.9% decrease in migration after 24 h for KE-3 p38δ and p-p38δ cells respectively compared to non-transfected cells (Fig. 5A). In addition p38δ and p-p38δ induced a significant decrease in KE-3 migration at 24 h [55.65±1.5 and 75.65±0.3% (p<0.001), respectively] and 48 h [37.9±0.8, (p<0.01) and 82.7±1.4%, (p<0.001) respectively] compared with non-transfected KE-3 cells using a wound healing assay (Fig. 5B and C). Finally, to further examine the influence of p38δ and p-p38δ on the growth characteristics of KE-3 cells, we measured their ability to grow in an anchorage-independent manner. Non-transfected KE-3 cells growing in soft agar for 21 days gave rise to 175±18 colonies/plate (Fig. 5D). This was similar to the number of colonies/plate that grew for cells transfected with empty vector (160±20) or p-p38δ_DN_ (177±21) (Fig. 5D). In contrast, however, p38δ and p-p38δ transfected cells produced a significant (p<0.001) decrease in colony numbers in p38δ transfected cells (13±3) with no colonies observable for p-p38δ transfected cells (Fig. 5D).

## Discussion

Oesophageal cancer is a highly aggressive treatment-refractory disease with a high mortality rate (2,5,6). As conventional therapy is ineffective, targeting specific potential molecular tumour markers may prove to be the future of oesophageal cancer treatment. Despite current studies of molecular targets in oesophageal cancer (36), we are still somewhat hindered by limited knowledge of the genes and pathways involved in the tumourigenesis of the oesophagus when it comes to treatment.

Emerging role(s) for p38 MAPKs in different aspects of cancer has recently been outlined. To-date the best studied and reviewed isoform in cancer is p38α. It has been characterized as both a potential tumour suppressor (25,37–39) and tumour promoter (29,35). In comparison the role(s) of p38δ in cancer is largely uncharacterised. The limited current knowledge pertaining to p38δ, however, also alludes to disparate role(s) for this kinase in tumour development. An oncogenic role for p38δ has been suggested in p38δ-deficient mice that have reduced susceptibility to skin carcinogenesis (40) as well as promoting head and neck squamous carcinoma cell growth (29). In contrast a very recent study outlined a role for p38δ as a tumour suppressor in mouse fibroblasts (41). In our study outlined here we show for the first time the differential expression of p38δ in OESCC cell lines and *in vivo*. The loss of p38δ expression provides a survival advantage for OESCC which demonstrates increased cell proliferation, migration and contact inhibition. Re-introduction of p38δ, however, leads to reversal of these tumourigenic effects. Thus, recent evidence (41) as well as our present study suggests that targeting p38δ may offer a powerful protection against carcinogenesis. Targeting p38 MAPK isoforms or pathways for therapeutic purposes, however, should perhaps be strictly dependent on cell context, tumour cell type and tumour stage.

The fusion of p38δ to its upstream kinase MKK6b or active MKK6b [MKK6b(E)] generated a constitutively active p38δ which was used as a tool to study its specific effect(s) in OESCC. Re-introduction of p38δ (with subsequent activation) or active p-p38δ into KE-3 OESCC attenuated cell proliferation, migration and anchorage-independent growth. The strength and duration of p38 activation has been shown to play a crucial role in determining cell fate. Strong activation has been shown to induce apoptosis whereas lower levels results in cell survival (27,39). In our study we observed strong anti-proliferative, anti-migratory effects as well as effects on anchorage-independent growth upon re-introduction of p38δ into KE-3 cells which subsequently became active. These antitumourigenic effects were amplified further in KE-3 cells transfected with constitutively active p-p38δ. It is possible that owing to the localization of both p38δ and p-p38δ in the nucleus and the cytoplasm of OESCC that this kinase may modify its target(s) either structurally or subcellularly. We are presently researching whether they are in free form or docked with specific cytoplasmic or nuclear partners (24). Furthermore, p38δ and p-p38δ induced antitumourigenic effects in OESCC may arise by a combination of both phosphorylation-dependent and independent effects as previously described (33,34).

There are many paradigms in the literature of cross-talk between different MAPK pathways. In this instance, however, when KE-3 cells were stably transfected with p38δ or p-p38δ we did not observe changes in either p38 isoform (α, -β and -γ), ERK1/2 or JNK1/2/3 expression (data not shown) or activation levels. This is in agreement with a recent bio-informatics analysis of MAPK pathways which specifically identified that persistent activation of p38δ is resistant to interaction with other MAPKs (42). This lack of interference from other MAPKs permits us to specifically study the effects of p38δ on cell cycle control, pathway components and regulatory mechanisms in OESCC which is currently ongoing in our laboratory. In addition negative feedback mechanisms have been shown to contribute to fine-tuning p38 MAPK activity levels. One such report outlines an increase in MKK6 expression and stability in p38α^−/−^ cardiomyocytes from transgenic mice (43). We did not observe a correlation between the presence or absence of p38δ expression in OESCC cells and MKK expression. Of notable exception is MKK3 whose expression is absent from KE-3 and -8 cells (both negative for p38δ) but present in KE-4, -5, -6 and -10 cells (all positive for p38δ). However, this pattern of expression does not hold for KYSE-70 and OE-21 OESCC cell lines which express MKK3 but are also negative for p38δ protein expression.

Reports of the involvement of p38 MAPKs in a variety of different pathological conditions is continuing to increase fuelling interest in the development of potent and specific drugs for modulating the activity of these kinases. Presently there are a number of p38 inhibitors undergoing clinical trials for the treatment of inflammatory diseases (44,45). Results arising from our study demonstrate that loss of p38δ expression in OESCC provides a more sinister phenotype with increased proliferation, migration and anchorage-independent growth. Thus, it is possible that isoform specific activation (rather than inhibition) of p38δ may provide a therapeutic benefit for patients with OESCC which express this isoform. In addition, how p38δ activators may interact and enhance the effectiveness of traditional therapeutics in combination therapy warrants attention.

In conclusion, our results reveal previously undocumented p38δ differential expression and function in OESCC. We identified a subset of OESCC cell lines as well as human primary and metastatic tumour samples that exhibit downregulation of p38δ protein expression. We now provide evidence that loss of expression of this particular isoform may be a mechanism by which OESCC cells promote carcinogenesis. Re-introduction of p38δ into OESCC negative cell lines suppressed different aspects of tumourigenesis. Our data warrant further investigation to understand the important physiological and pathophysiological effects of p38δ in OESCC and is currently in progress. This knowledge should identify which pathways, substrates or regulators are affected specifically by p38δ in providing an antitumourigenic effect in OESCC. Armed with this information uncovering novel targets and the development of new therapeutics may be possible for this common cancer that continues to demonstrate a generally poor clinical outcome.

## Figures and Tables

**Table I:** Patient characteristics, and cell lines used.

A, OESCC patient features	

Patient features	No. of patients
Gender	
Male	4
Female	6
Age, median (years)	63 (44–81)
TNM7 stage	
T stage	
T3	10
N stage	
N1	3
N2	7
Histological grade	
Well differentiated	1
Moderately differentiated	6
Poorly differentiated	3

B, KE (OESCC) cell line features	

KE features	
Gender	
Male	KE-3, -4, -5, -6
Female	KE-8, -10
Age, median (years)	67 (50–71)
TNM7 stage	
T stage	
T1	KE-10
T3	KE-3, -5, -6, -8
T4	KE-4
N stage	
N0	KE-5
N1	KE-3, -4, -6, -8, -10
Histological grade	
Well differentiated	KE-5, -6
Moderately differentiated	KE-3, -10
Poorly differentiated	KE-4, -8

C, p38δ MAPK expression in patient specimens		

Diagnosis	p38δ MAPK expression	
	Positive	Negative
NAT (n=10)	9	1
OESCC primary (n=10)	6^a^	4
OESCC nodes (n=10)	2	8

A, Patient features and B, KE features are summarised based on gender, age, TNM7 stage and histological stage. Based on the TNM7 categorization for oesophageal cancer N1=1–2 lymph nodes and N2=3–6 lymph nodes. C, Samples obtained from ten patients consisted of ten paired primary tumour and metastatic lymph nodes as well as corresponding non-tumour adjacent tissues for analysis of p38δ expression.

a p38δ expression was considerably lower than in the corresponding NAT.
